# C-Lobe of Lactoferrin: The Whole Story of the Half-Molecule

**DOI:** 10.1155/2013/271641

**Published:** 2013-05-15

**Authors:** Sujata Sharma, Mau Sinha, Sanket Kaushik, Punit Kaur, Tej P. Singh

**Affiliations:** Department of Biophysics, All India Institute of Medical Sciences, New Delhi 110029, India

## Abstract

Lactoferrin is an iron-binding diferric glycoprotein present in most of the exocrine secretions. The major role of lactoferrin, which is found abundantly in colostrum, is antimicrobial action for the defense of mammary gland and the neonates. Lactoferrin consists of two equal halves, designated as N-lobe and C-lobe, each of which contains one iron-binding site. While the N-lobe of lactoferrin has been extensively studied and is known for its enhanced antimicrobial effect, the C-lobe of lactoferrin mediates various therapeutic functions which are still being discovered. The potential of the C-lobe in the treatment of gastropathy, diabetes, and corneal wounds and injuries has been indicated. This review provides the details of the proteolytic preparation of C-lobe, and interspecies comparisons of its sequence and structure, as well as the scope of its therapeutic applications.

## 1. Lactoferrin: A Bilobal Protein

**Figure 1:**
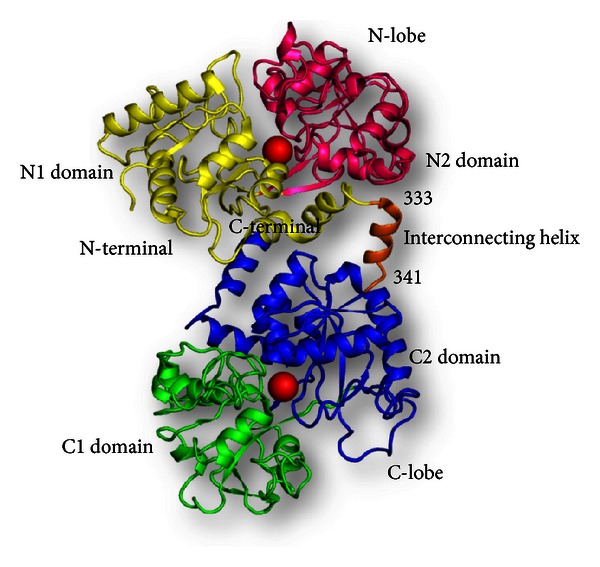
Schematic diagram of the bovine lactoferrin molecule (PDB code: 1BLF). The N1 and N2 domains are colored in yellow and pink, respectively, while the C1 and C2 domains are colored in green and blue, respectively. The interconnecting helix between the lobes is colored in orange. The two iron atoms are shown as red spheres.

**Figure 2:**
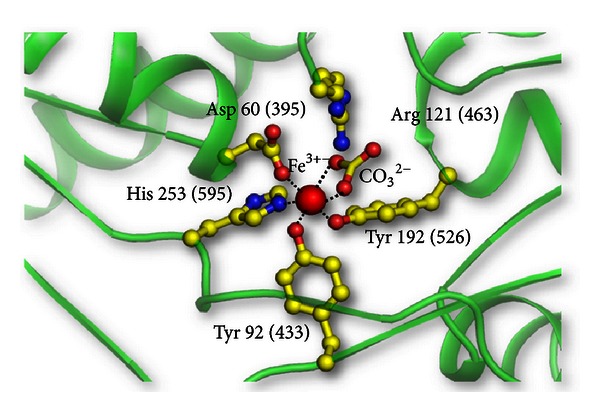
Schematic figure of the iron-binding site of lactoferrin. The iron atom is shown as a red sphere, while the interacting amino acid residues of lactoferrin are in yellow. The residue numbers correspond to N-lobe, while the corresponding residues of C-lobe are in brackets.

Lactoferrin is an iron-binding glycoprotein which is abundantly present in colostrum and is the major mediator of defence for the newborn [1–4]. The three-dimensional structures of lactoferrin from various species, such as human [5, 6], bovine [7, 8], buffalo [9, 10], equine [11, 12], and camel [13] have been solved. In all these cases, the overall structures of iron-saturated lactoferrin were found to have a similar folding. Lactoferrin is an 80 kDa single-chain glycoprotein which can be equally divided into two homologous halves, N-lobe and C-lobe, each of which is about 40 kDa in size and is connected to the other by a short helical segment (Figure 1). Each lobe is further subdivided into two domains, which are designated as N1 and N2 domains of N-lobe and C1 and C2 domains of C-lobe. The iron-binding site is situated inside the interdomain cleft in each lobe. The iron-binding site consists of four residues 2 tyrosines, 1 aspartate, and 1 histidine residues. The iron-binding residues in N-lobe are Asp 60, Tyr 92, Tyr 192, and His 253 while the corresponding iron-binding residues in C-lobe are Asp 395, Tyr 433, Tyr 526, and His 595. The iron-binding residues are coordinated to the ferric ion and a synergistic bidentate carbonate anion (Figure 2). 

Though there has been a considerable amount of information on the structure of native lactoferrin, a similar knowledge on the structures of two independent lobes upon cleavage of lactoferrin has been scarce. Hence, there have been efforts since the discovery of lactoferrin to uncouple the two lobes using limited proteolysis and thereby study their structures and associated functions. 

## 2. Preparation of Monoferric Lobes of Lactoferrin: Early Days to Present Times

The earliest reports of the production of C-lobe came in 1976 when both iron-saturated and apolactoferrin were digested with trypsin [14]. It was found that the iron-saturated lactoferrin was more resistant to proteolysis than apolactoferrin. However, instead of generating two equal 40 kDa lobes, five different fragments with molecular weights ranging from 25,000 to 52,700 were produced.

Later reports of proteolysis of lactoferrin with trypsin did not report success on the generation of two equal halves of lactoferrin. In fact, it was established that proteolysis of iron-saturated human lactoferrin with trypsin generates a 30 kDa N-terminal fragment and a 50 kDa C-terminal fragment. Upon further hydrolysis of N-lobe with a second tryptic digestion, the ND2 domain of 18 kDa was generated [15]. 

On the other hand, the first report on the human N-lobe produced from the cloned cDNA expressed in baby hamster kidney (BHK) cells appeared [16]. Iron binding and release studies on this recombinant N-lobe showed that the absence of stabilizing contacts between the N- and C-lobes led to faster and easier release of iron from the N-lobes as compared to that from native lactoferrin. Similarly, C-lobe of human lactoferrin was also cloned and expressed in due time [17]. 

The first report on the generation of equal 40 kDa N-lobe and C-lobe came in 1999, where it was reported that limited proteolysis of buffalo lactoferrin with proteinase K, a nonspecific serine protease, led to the production of two 40 kDa equal-sized lobes along with low molecular mass peptides (<14 kDa) [18]. The proteolysis of buffalo lactoferrin with trypsin and pepsin produced two major fragments of approximately 35 and 23 kDa together with small molecular mass peptides. Subtilisin, another enzyme, hydrolyzed lactoferrin to produce two fragments of 40 and 26 kDa along with low molecular mass peptides [18]. N-lobe and C-lobe, generated by proteolysis with proteinase K, were further purified using ion-exchange and gel filtration chromatography. Upon further hydrolysis, it was found that N-lobe was completely digested into low molecular mass peptides, while C-lobe remained intact for a long time indicating it to be significantly resistant to digestion by proteases. The resistance of C-lobe to proteolysis was attributed to a peptide found within the C-lobe, which bound with and hence inhibited proteinase K. 

The secondary structure elements of both the purified lobes and the native lactoferrin were analyzed by circular dichroism studies. It was found that lower helical structures were present in the N- and C-lobes as compared to native lactoferrin [18]. Similarly, the iron saturations of the native lactoferrin was found to be higher than that of N- and C-lobes. 

Another report on the cloning and expression of C-lobe followed where a *Rhodococcus erythropolis* expression system for the bovine lactoferrin C-lobe was constructed which yielded a native and a denatured form of C-lobe [19]. However, the denatured protein had to be refolded by stepwise dialysis against refolding buffers in order to generate a bioactive, folded C-lobe. After almost three decades of work on the generation of the N-lobe and C-lobe which included both the proteolysis as well as cloning efforts, the proteolysis of lactoferrin with proteinase K was the most effective way, as it also led to the crystallization of C-lobe and to its successful structure determination [20]. The structure of cloned C-lobe has not been determined to date. 

## 3. Structure of C-Lobe within Native Lactoferrins

The structures of lactoferrin were solved in iron-saturated form from five species, equine [11] (PDB Code: 1B1X), human [5] (PDB Code: 1LFG), bovine [7, 8] (PDB Code: 1BLF), and buffalo [9, 10] (PDB Code: 1BIY), while the structures of iron-free (apo) forms of lactoferrin could be determined only from three species, equine [12] (PDB Code: 1B7U), human [6] (PDB Code: 1CB6), and camel [13] (PDB Code: 1DTZ). Within the structure of native lactoferrin in all species, the C-lobe was found to have a two-fold internal homology with N-lobe, and both the lobes were connected by a short helical segment. 

**Figure 3:**
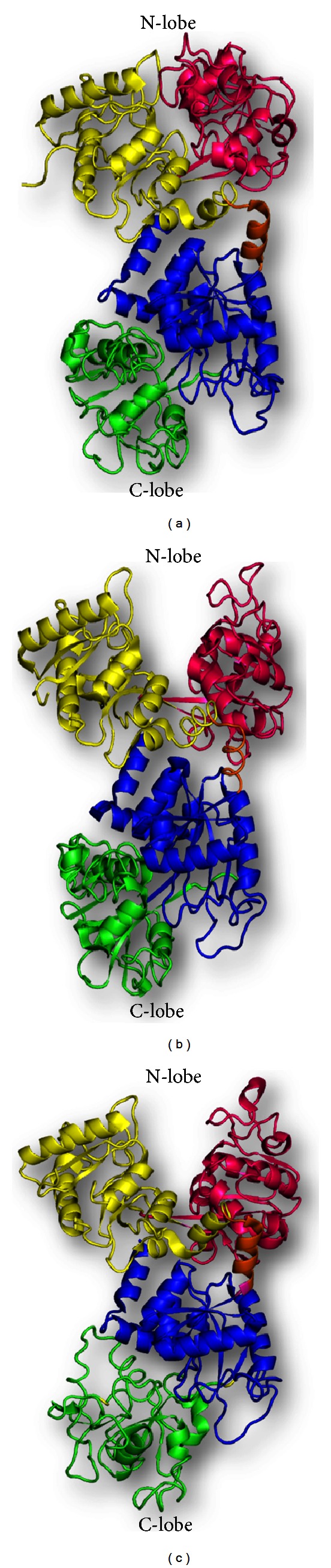
Schematic diagrams of apolactoferrins from three species showing variable behavior of the conformation of the domains. (a) Equine apolactoferrin (PDB code: 1B7U) shows both the lobes in closed conformation. (b) Human apolactoferrin (PDB code: 1CB6) shows open N-lobe and closed C-lobe. (c) Camel apolactoferrin (PDB code: 1DTZ) shows both the lobes in open conformation.

 In the iron-saturated forms of lactoferrin, both the N-lobe and C-lobe adopt a closed conformation [5, 7–11], though there were differences in the degree of closure of the two domains of each lobe in each species. However, in the case of apolactoferrin, the N-lobe and C-lobe showed differential conformations depending on the species from which the lactoferrin originated. In case of apoequine lactoferrin (AELF), both the N- and C-lobes adopt the closed conformation [12] (Figure 3(a)). In case of human apolactoferrin (AHLF), the N-lobe adopts the open conformation while C-lobe remained in the closed form [6] (Figure 3(b)). However, in the case of apocamel lactoferrin (AULF), both the N-lobe and C-lobe were found in open conformations, showing wide distances between the iron-binding residues in the native iron-free form of lactoferrin [13] (Figure 3(c)).

The differences in the conformations of N-lobe of apolactoferrins from three different species were explained by the presence of four crucial interactions at the mouth of the iron-binding site in the N-lobe of equine lactoferrin, which were absent from other lactoferrins [11, 12]. The first interaction was a hydrogen bond between Ser12 in N1 domain and Ser185 in N2 domain which brought the two domains together. The second interaction which was responsible for the closure of N-lobe was a salt bridge between Lys301 and Glu216. Also, the interactions between Arg249*⋯*Gln89 and Asp211*⋯*Gln83 are also responsible for the N-lobe to remain closed in the apostate in AELF [12].

While the C-lobes of both equine and human lactoferrins remain closed in the apostate, the C-lobe of camel lactoferrin is observed to be in the open conformation. This was explained by the absence of one interaction in camel apolactoferrin which was present in equine and human lactoferrins. This occurs due to differences in the sequence of camel lactoferrin, as compared to other lactoferrins. In camel lactoferrin, Gln418 is replaced by Pro418, and hence the crucial interaction between Gln418 with Arg587 (human lactoferrin) and Gln587 (equine lactoferrin) is missing. 

It was also seen that the positioning and conformations of residues of the iron-binding site of N-lobe of camel apolactoferrin were similar to those of the N-lobe in human apolactoferrin. However, the corresponding residues in the C-lobe were similar to those in the C-lobes of duck and hen apoovotransferrins [13]. This indicated that in terms of structure and iron binding and release, while the N-lobe of camel apolactoferrin is similar to the N-lobe of human apolactoferrin, the C-lobe of camel apolactoferrin, however, showed similarity with the C-lobe of hen and duck apoovotransferrins. These findings were validated by performing iron binding and release studies on the proteolyzed, independent lobes, where it was seen that the N-lobe of camel lactoferrin loses iron at a low pH (4.0–2.0), which matched with other lactoferrins. On the other hand, the iron-binding and iron-release behaviors of the C-lobe of camel lactoferrin is similar to that in transferrins, as it loses iron at a much higher pH of 6.0 and above [13]. 

**Figure 4:**
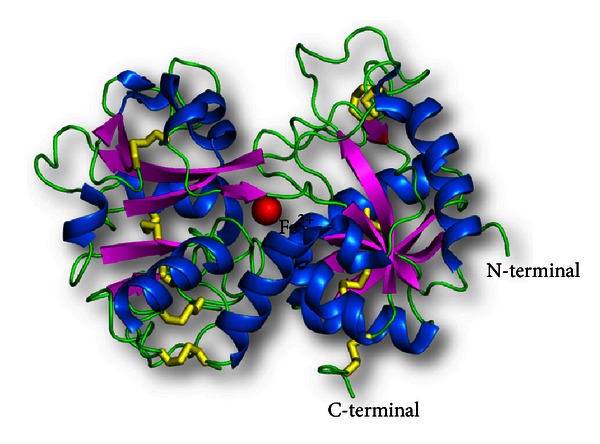
Schematic diagrams of C-lobe of bovine lactoferrin, produced using limited proteolysis with proteinase K (PDB code: 1NKX). *α*-helices are represented as blue helices, *β*-sheets are indicated by magenta arrows, and disulfide bonds are indicated as yellow sticks. The iron atom is shown in red.

The first crystal structure of a proteolytically generated functional iron-saturated C-lobe of bovine lactoferrin (Fe-C-Lobe) revealed 2593 protein atoms (residues 342–676 and 681–685), 124 carbohydrate atoms (from ten monosaccharide units, in three glycan chains), one Fe^3+^, one CO_3_^2−^, two Zn^2+^, and 230 water oxygen molecules [20] (Figure 4).

The structure of bovine C-lobe began from residue 342 and ended with residue 676, with a cleaved pentapeptide fragment 681–685 which remained attached to the C-lobe through a disulphide bridge, Cys405–Cys684. Though the overall folding of the C-lobe was found to be similar to that of C-lobe in the native bovine lactoferrin, there were interesting differences in the conformations of some of the loops. It also showed the presence of a number of novel interactions which seemed to have been generated to stabilize the structure after proteolysis and loss of stabilizing interaction with the N-lobe. The secondary structure topology diagram of Fe-C-Lobe is depicted in Figure S1 in supplementary material available online at http://dx.doi.org/10.1155/2013/271641.

The most interesting observation was the presence of 6 interdomain hydrogen bonds in the structure of Fe-C-Lobe, which was more than the 4 interdomain hydrogen bonds reported in the structure of Fe-Lf [20]. Two zinc ions, which were found to be situated at sites other than the iron-binding cleft, seemed to be significant in providing stability to the crystal packing. 

**Figure 5:**
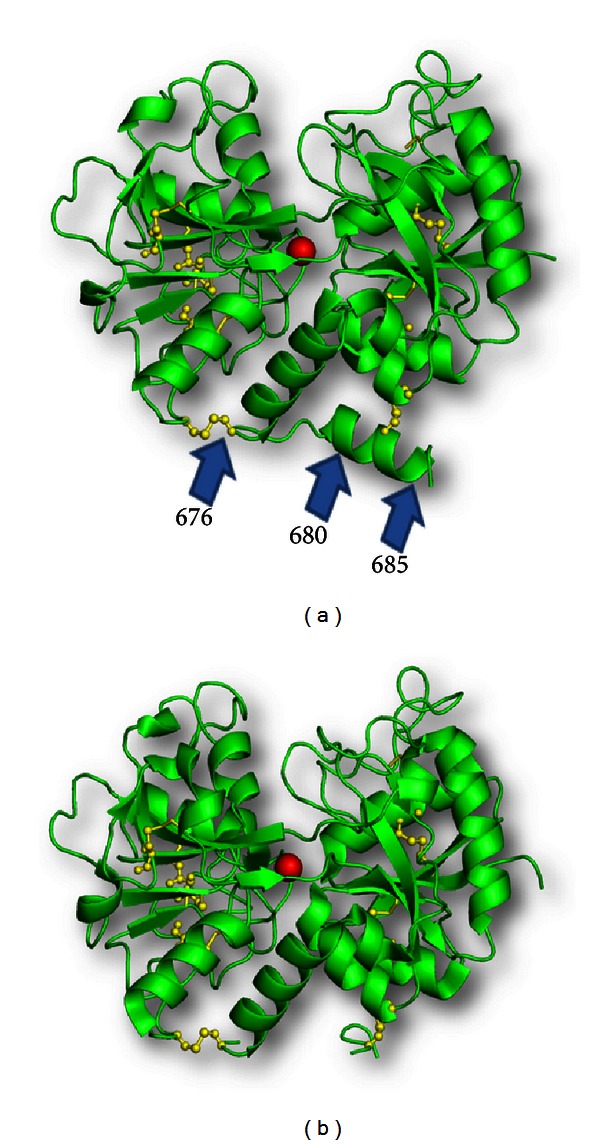
A comparison of the (a) C-lobe of intact lactoferrin and (b) C-lobe which has been cleaved from the intact lactoferrin using proteinase K. Apart from one single cut at the N-terminus, the enzyme cleaves the C-terminal part of the protein at three points, leading to the generation of a pentapeptide which is anchored to the main protein by a disulfide bond between C405 and C684.

The action of proteinase K seemed to be on the most exposed two protein segments 334–344 and 674–689, both of which cross over from the N-lobe to the C-lobe and from the C-lobe to the N-lobe, respectively. These two segments are responsible for providing interlobe interactions between the two lobes in lactoferrin. Hence, many interactions were observed to be lost due to cleavage of N- and C-lobes, leading to a rearrangement of side chains of C-lobe in the absence of interacting N-lobe side chains. The C-terminus of Fe-C-Lobe showed that Proteinase K had cleaved the lactoferrin at three points at the C-terminus, Ser676, Leu680, and Ala 685, leading to a loss of helix, designated as *α*12 in native lactoferrin, and generation of pentapeptide loop, that is, still anchored to the protein by a disulfide bond between Cys684 and Cys405 (Figure 5). In conclusion, the structure of C-lobe, starting from residue 342 and ending with residue 676, with a pentapeptide, 681–685 anchored to the main structure, was a fairly stable structure which was able to bind and release iron and also perform other functions.

## 4. C-Lobe of Lactoferrin: Sequence Comparison

The sequence comparison of bovine C-lobe with the corresponding N-lobe showed 30% sequence identity between the two lobes (Figure S2). Three C-lobe fragments >5 residues were found to be absent in the N-lobe. The three fragments were: Ser-Ser-Leu-Asp-Cys-Val-Leu-Arg, Thr-Asn-Gly-Glu-Ser-Thr-Ala-Asp, and the C-terminal fragment, Ala-Cys-Ala-Phe-Leu-Thr-Arg. 

Interestingly, all these three fragments have a functional significance within the C-lobe. The first two fragments have been found to bind to the HA(2) region of viral hemagglutinin, an action which is not performed by N-lobe [21]. The third fragment, the highly hydrophobic C-terminal fragment, is the peptide, that is nicked by proteinase K [20] and is found to be attached to the C-lobe molecule by a disulfide bond even after its cleavage by the nonspecific proteinase K, indicating that it may also have a functional relevance in the antimicrobial role of lactoferrin. 

**Figure 6:**
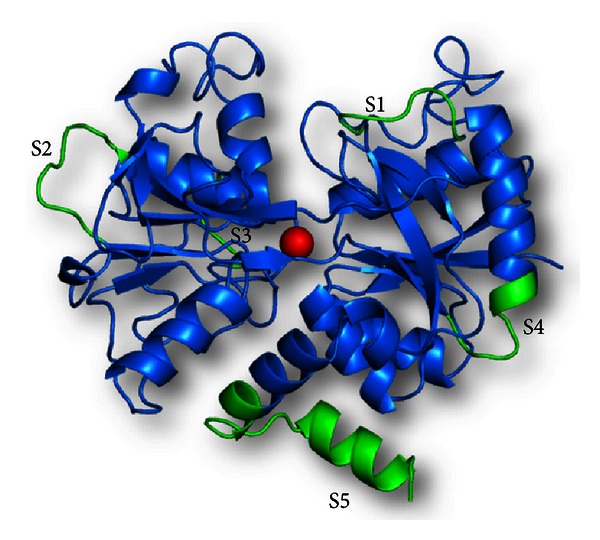
Schematic diagrams of C-lobe of bovine lactoferrin (in blue), showing the sequences which are most variable across species in green. The sequences have been labeled S1–S5.

The sequence comparison of bovine C-lobe with the corresponding C-lobe of buffalo, caprine, camel, human, equine, and porcine milk revealed a sequence identity of 96%, 95%, 74%, 73%, 72%, and 72%, respectively. There were at least 5 stretches of sequences >5 residues that were found to be remarkably different from each other (Figure S3). They corresponded to sequence Ser-Lys-His-Ser-Ser-Leu, Lys-Lys-Ala-Asn-GluGly-Leu, Asp-Asp-Gln-Gly-Leu-Asp, Arg-Ser-Asp-Arg-Ala-Ala-His-Val-Glu-Gln, and Ala-Asn-Leu-Lys-Lys-Cys-Ser-Thr-Ser-Pro-Leu-Leu-Glu-Ala-Cys-Ala-Phr-Leu-Thr-Arg, designated as S1, S2, S3, S4, and S5, respectively (Figure 6).

All these sequences were found to be flexible loops exposed to the surface, while the core of the C-lobe remains constant, indicating that there has been an independent evolution of the core and the exposed regions of C-lobe. S1 and S2 were part of the sequences that bind to viral hemagglutinin [22] and are absent in N-lobe. These loops are highly variable in C-lobe across species and are absent in N-lobe, indicating that these sequences could be specific to C-lobe of lactoferrin but seem to have evolved with the C-lobe for a functional need. In case of porcine lactoferrin, S5, the C-terminal sequence starting from residue 673 is entirely missing. Interestingly, this is the longest sequence among the most variable sequences ofC-lobe and is also the same that is found anchored to the C-lobe after cleavage by proteinase K, indicating that there has been a structural evolution of C-terminus of this protein, and it may be required for some specialized function, since it remains anchored to the protein despite cleavage by an aggressive protease. 

## 5. Therapeutic Applications of C-Lobe

**Figure 7:**
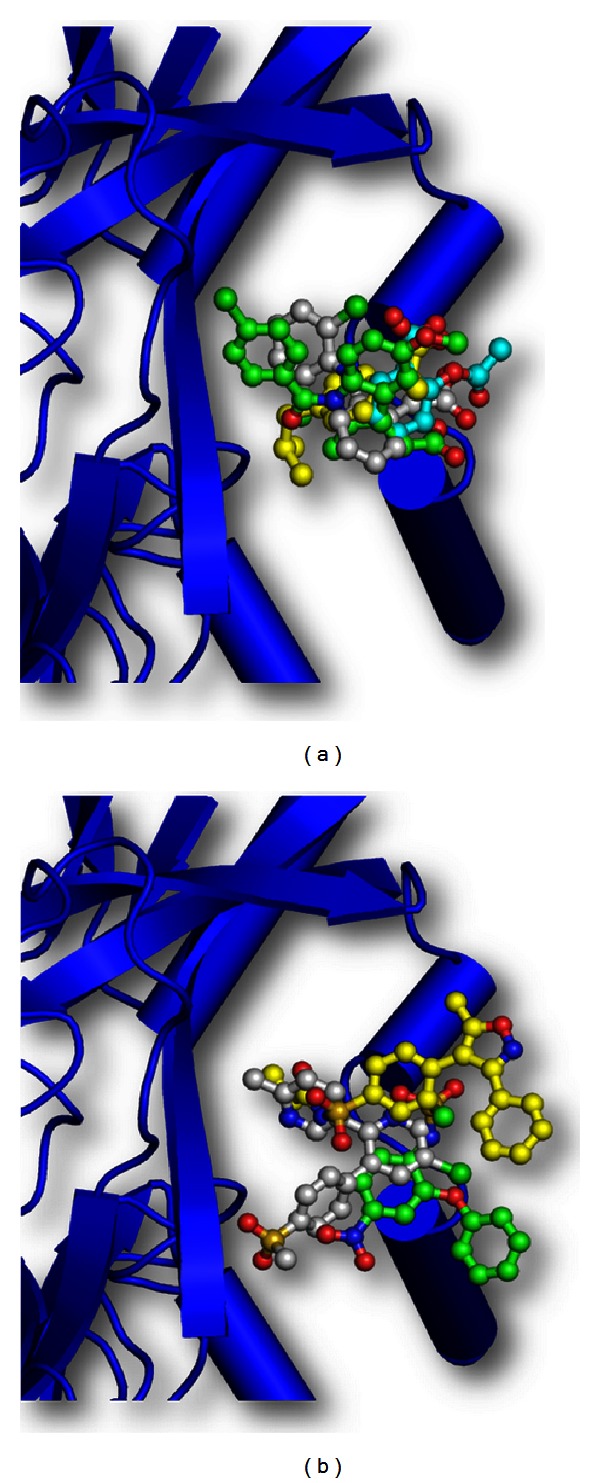
Schematic diagram of complex of C-lobe of lactoferrin with (a) nonsteroidal anti-inflammatory drugs (NSAIDS), aspirin (cyan), diclofenac (white), indomethacin (green), and ibuprofen (yellow) and (b) COX-2 specific inhibitors, etoricoxib (white), parecoxib (yellow), and nimesulide (green).

C-lobe of bovine lactoferrin was found to exert a protective role in the prevention of nonsteroidal anti-inflammatory drug- (NSAID-) induced gastropathy [23]. The structural basis of this action of C-lobe was discovered after observing the bindings of NSAIDs to C-lobe and by determining the structures of complexes of bovine C-lobe with four commonly prescribed NSAIDs, indomethacin, diclofenac, aspirin, and ibuprofen, as well as COX-2 specific NSAIDs (Figure 7(a)). This therapeutic action of C-lobe was also detected in mouse models, where it was found that while the addition of C-lobe led to the effective reversal of NSAID-induced gastropathy, its coadministration with NSAIDs prevented it significantly [23]. This effect was mediated by a novel drug-binding site in lactoferrin which effectively sequesters the unbound NSAIDs in the gut. A similar study of C-lobe with COX-2 specific NSAIDs showed that the C-lobe was able to bind and sequester COX-2 specific NSAIDs with the same efficiency (Figure 7(b)) [24].

As observed by the binding studies (Table 1) [24, 25] and the structure determination studies, the same binding site in C-lobe also functions as a binding site for sugars. C-lobe has also been shown to reduce the levels of glucose due to its binding with various edible sugars [25] which bind at the sugar-binding site in the protein. While the administration of C-lobe to human serum showed reduction in the glucose levels, it was shown to be mediated by the binding of C-lobe with various sugar molecules, such as glucose, galactose, mannose, xylose, maltose, cellobiose, lactose, sucrose, and dextrin. All the sugars bound to the sugar-binding site of the C-lobe with varying potencies ranging around 10–4 M but were chelated by the C-lobe effectively. It may be mentioned here that C-lobe sequesters the NSAIDs only temporarily to release them gradually on demand as the binding affinities to these compounds vary between 100 micromolar to 1 millimolar. In fact, here the role of C-lobe is to protect the mucus of the intestinal lining from the interactions of NSAIDs that cause injury to tissues. In order to prevent NSAID-induced gastropathy, it is important that the target molecule should bind to the NSAIDs with low affinity, since if it binds with a high affinity, it would compete with specific enzymes such as cyclooxygenases in the gut for NSAID molecules which would reduce the efficacy for NSAIDs and defeat the purpose of these drugs. Hence, the administration of NSAIDs with the coadministration of C-lobe would be the right strategy for alleviating NSAID-induced gastropathy, as this would ensure that NSAIDs could bind to cyclooxygenases in the gut and reduce inflammation, and yet the excess of NSAIDs would be chelated by the C-lobe, so that damage to the gut could be reduced. For this purpose, the moderate binding affinities as found between C-lobe and NSAIDs are appropriate. 

**Figure 8:**
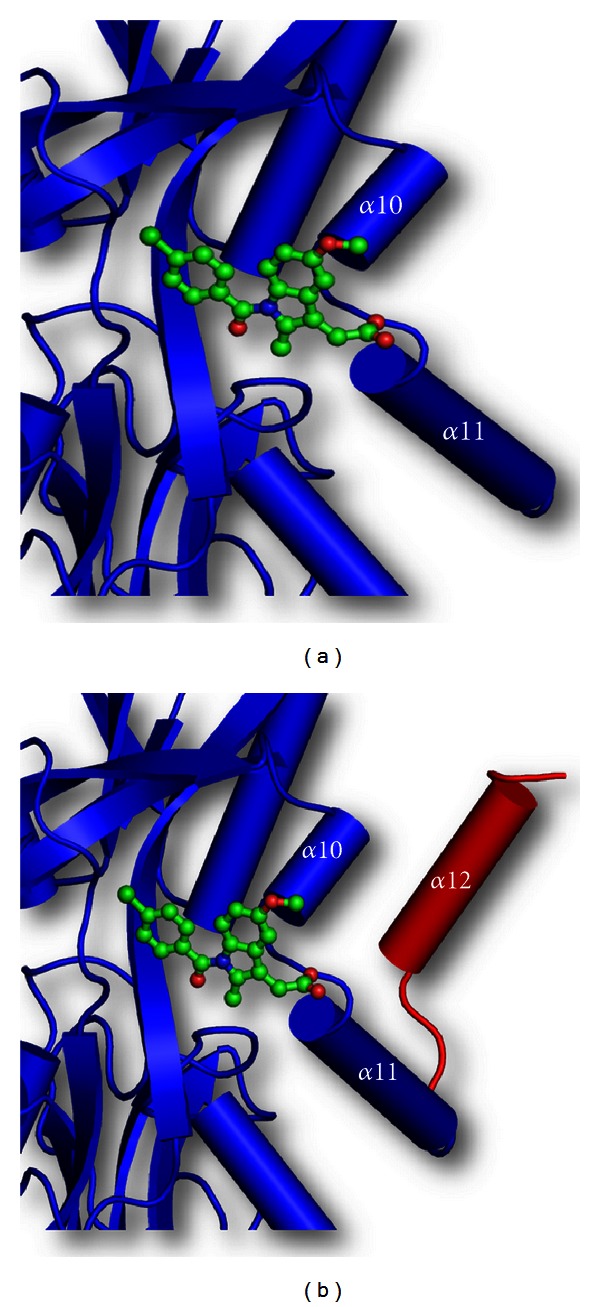
Schematic diagram of complex of (a) C-lobe of lactoferrin (blue) with indomethacin (green), showing the accessible drug-binding site in between the two helices, *α*10 and *α*11. The *α*12 helix has been cleaved by proteinase K to make this site more accessible and (b) C-lobe of lactoferrin (blue) with indomethacin (green), with superimposed *α*12 helix (in red) from native bovine lactoferrin, which shows that this helix blocks the passage to the binding site.

In this context, it may be mentioned here that NSAID and sugar-binding site in lactoferrin is slightly obstructed by the C-terminal helix, *α*12 which is removed from the C-lobe by Proteinase K on proteolysis, making this functional site more accessible (Figure 8). This makes the C-lobe a superior molecule against gastropathy caused by NSAIDs than the intact lactoferrin. 

C-lobe was shown to have a role in anti-influenza therapeutics as it showed inhibition of influenza virus hemagglutination and cell infection, which the N-lobe was not able to perform [22]. This action of C-lobe was attributed to three C-lobe fragments which strongly bind to the HA(2) region of viral hemagglutinin which is the highly conserved region containing the fusion peptide.

Bovine C-lobe was also found to be effective in the corneal epithelial wound closure in vitro at concentrations of ≥6.4 *μ*M. On comparison of this effect with the native BLF or N-lobe, C-lobe was almost twice as effective which indicated that BLF C-lobe may be a novel treatment for corneal lesions with delayed healing [26]. 

In another study, the C-lobe of camel lactoferrin was found to have antiviral activity against hepatitis C virus in a study which involved evaluation of inhibitory effects on hepatitis C virus into Huh7.5 cells after incubation of hepatitis C virus with the C-lobe of lactoferrin prior to infection [21]. 

Bovine C-lobe was demonstrated to promote bifidobacterial growth despite the fact that it did not bind with surface proteins from bifidobacteria, unlike the N-lobe and native lactoferrin which showed binding to those proteins, indicating that C-lobe may be enhancing bifidobacterial growth using mechanisms other than direct binding with the surface proteins from bifidobacteria [27, 28]. 

Upon investigating the effects of the two lobes of bovine lactoferrin on the contractile activity of collagen gels, it was found that the C-lobe had a greater effect on collagen gel contractile activity as compared to native bovine lactoferrin or its N-lobe [29]. While this effect of C-lobe was found to be markedly dose dependent, it was completely abolished upon further hydrolysis of the C-lobe with serine proteases like pepsin or trypsin. In another study, using in-gel ATPase activity assays, it was shown that the nucleoside-5′-triphosphate-hydrolyzing activity of lactoferrin is found in the C-lobe [30]. 

## 6. Conclusions

Lactoferrin is a multifunctional protein that mediates its various functions using its two molecular halves N-lobe and C-lobe. Though structurally, both the lobes are organized in a similar way, they contain certain stretches of unique sequences, which are significant functionally. N-lobe has been established as the lobe which is primarily involved in the antimicrobial function. On the other hand, C-lobe displayed varied therapeutic functions, due to which this lobe has a potential to be used for diseases like gastropathy, diabetes, and wound healing. The molecule of C-lobe produced proteolytically by cleaving the lactoferrin by proteinase K allowed the determination of its structure. The binding studies and the structure determinations of complexes of C-lobe with NSAIDs and sugars gave insights into the chemical properties and structural aspects of its functional properties. More studies need to done on the C-lobe of lactoferrin in order to harness it as a future drug.

## Supplementary Material

The supplementary material consists of three figures, Figures S1, S2 and S3. Fig. S1 depicts the secondary structure topology diagram of C-lobe of bovine lactoferrin. The *α*-helices are shown as red cylinders while the *β*-sheets are shown as green arrows. The C-terminus of the protein is irregular, with a pentapeptide hanging on with a disulfide bond. Fig. S2 depicts the sequence alignment of bovine N-lobe and bovine C-lobe. The residues have been numbered from 1 to 348. The sequence identity of both the lobes is 30%. The dashes at places indicate missing sequences. Identical sequences are shown in cyan. Fig. S3 depicts sequence alignment of C-lobe from various species of mammals, namely, Bovine C-lobe (CC-lobe), Buffalo C-lobe (BC-lobe), Caprine C-lobe (GClobe), Camel C-lobe (UC-lobe), Human C-lobe (HC-lobe), Equine C-lobe (EC-lobe) and Porcine C-lobe (PC-lobe). The residues have been numbered from 342 to 690. The dashes at places indicate missing sequences. Identical sequences are shown in cyan.

## Figures and Tables

**Table 1:** The binding constants of various NSAIDs and sugar molecules with the C-lobe of lactoferrin.

S. no.	Compound	Binding constant (10^−4^ M)
NSAIDs		
1	Indomethacin	2.6
2	Aspirin	3.3
3	Ibuprofen	4.8
Sugars		
1	Glucose	1.8
2	Galactose	1.7
3	Mannose	1.7
4	Xylose	1.8
5	Maltose	1.6
6	Cellobiose	1.7
7	Lactose	1.5
8	Sucrose	1.9
9	Dextrin	1.4
