# Supplementary figures and images for: C-Lobe of Lactoferrin: The Whole Story of the Half-Molecule
Source: Biochem Res Int. 2013 May 15;2013:271641. doi: 10.1155/2013/271641 (PMC3671519; doi:10.1155/2013/271641)

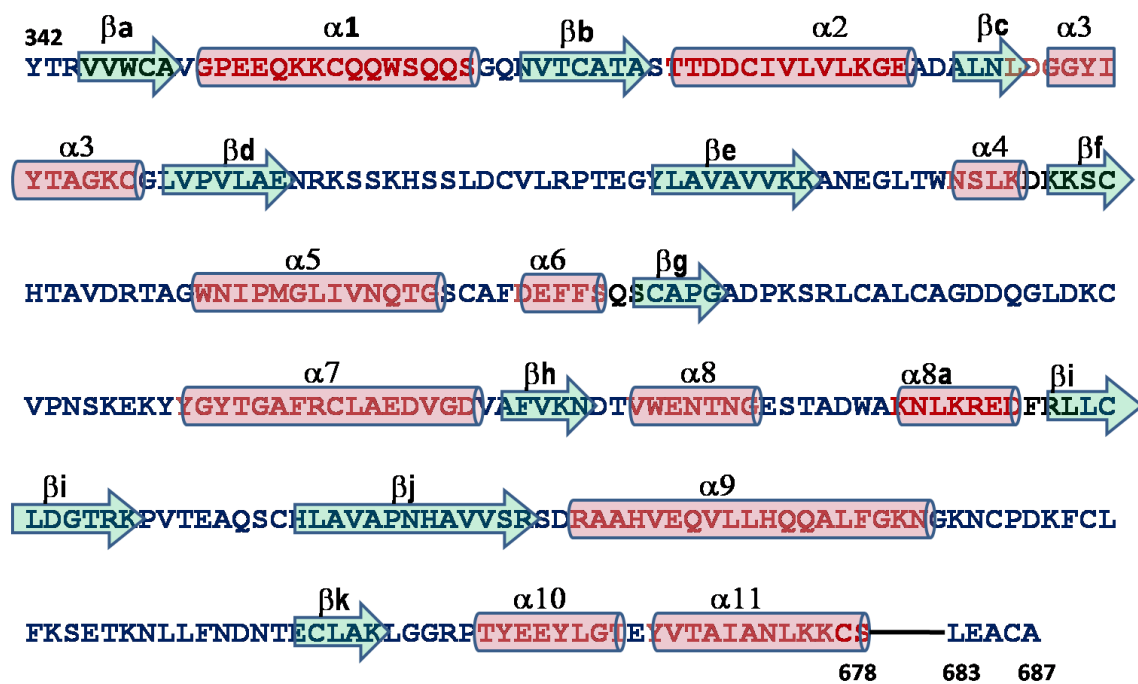

Fig. S1

Supplement: Supplementary file 1 — The supplementary material consists of three figures, Figures S1, S2 and S3. Fig. S1 depicts the secondary structure topology diagram of C-lobe of bovine lactoferrin. The α-helices are shown as red cylinders while the β-sheets are shown as green arrows. The C-terminus of the protein is irregular, with a pentapeptide hanging on with a disulfide bond. Fig. S2 depicts the sequence alignment of bovine N-lobe and bovine C-lobe. The residues have been numbered from 1 to 348. The sequence identity of both the lobes is 30%. The dashes at places indicate missing sequences. Identical sequences are shown in cyan. Fig. S3 depicts sequence alignment of C-lobe from various species of mammals, namely, Bovine C-lobe (CC-lobe), Buffalo C-lobe (BC-lobe), Caprine C-lobe (GClobe), Camel C-lobe (UC-lobe), Human C-lobe (HC-lobe), Equine C-lobe (EC-lobe) and Porcine C-lobe (PC-lobe). The residues have been numbered from 342 to 690. The dashes at places indicate missing sequences. Identical sequences are shown in cyan. [file 271641.f1.pdf]
