# Supplementary material for: C-Lobe of Lactoferrin: The Whole Story of the Half-Molecule
Source: Biochem Res Int. 2013 May 15;2013:271641. doi: 10.1155/2013/271641 (PMC3671519; doi:10.1155/2013/271641)
Supplement: Supplementary file 2 [file 271641.f2.pdf]

```
N-lobe     1   APRKNVRWCTISQPEWFKCRRWQWRMKKLGAPSITCVRRAFALECIRAIAEKKADAVTLD 60
C-lobe     342 --YTRVVWCAVGPEEQKKCQQWSQ----QSGQNVTCATASTTDDCIVLVLKGEADALNLD 395

N-lobe     61  GGMVFEAGRDPYKLRPVAAEIYGTKES--------PQTHYYAVAVVKKGSN-FQLDQLQG 111
Clobe      396 GGYIYTAG--KCGLVPVLAENRKSSKHSSLDCVLRPTEGYLAVAVVKKANEGLTWNSLKD 454

N-lobe     112 RKSCHTGLGRSAGWIIPMGILRPYLSWTESLEPLQGAVAKFFSASCVPCIDRQAYPNLCQ 171
C-lobe     455 KKSCHTAVDRTAGWNIPMGLIVN--------QTGSCAFDEFFSQSCAPGADPKSR--LCA 503

N-lobe     172 LCKGE--GENQCACSSREPYFGYSGAFKCLQDGAGDVAFVKETTVFEN--------LPEK 221
C-lobe     504 LCAGDDQGLDKCVPNSKEKYYGYTGAFRCLAEDVGDVAFVKNDTVWENTNGESTADWAKN 563

N-lobe     222 ADRDQYELLCLNNSRAPVDAFKECHLAQVPSHAVVARSVDGKEDLIWKLLSKAQEKFGKN 281
C-lobe     564 LKREDFRLLCLDGTRKPVTEAQSCHLAVAPNHAVVSRSD--RAAHVEQVLLHQQALFGKN 621

N-lobe     282 -KSRSFQLFGSPPGQRDLLFKDSALGFLRIPSKVDSALYLGSRYLTTLKNLRETAEEVKA 340
C-lobe     622 GKNCPDKFCLFKSETKNLLFNDNTECLAKLGGRPTYEEYLGTEYVTAIANLKKCSTSPLL 681
```

Fig. S2
