# Supplementary material for: C-Lobe of Lactoferrin: The Whole Story of the Half-Molecule
Source: Biochem Res Int. 2013 May 15;2013:271641. doi: 10.1155/2013/271641 (PMC3671519; doi:10.1155/2013/271641)
Supplement: Supplementary file 3 [file 271641.f3.pdf]

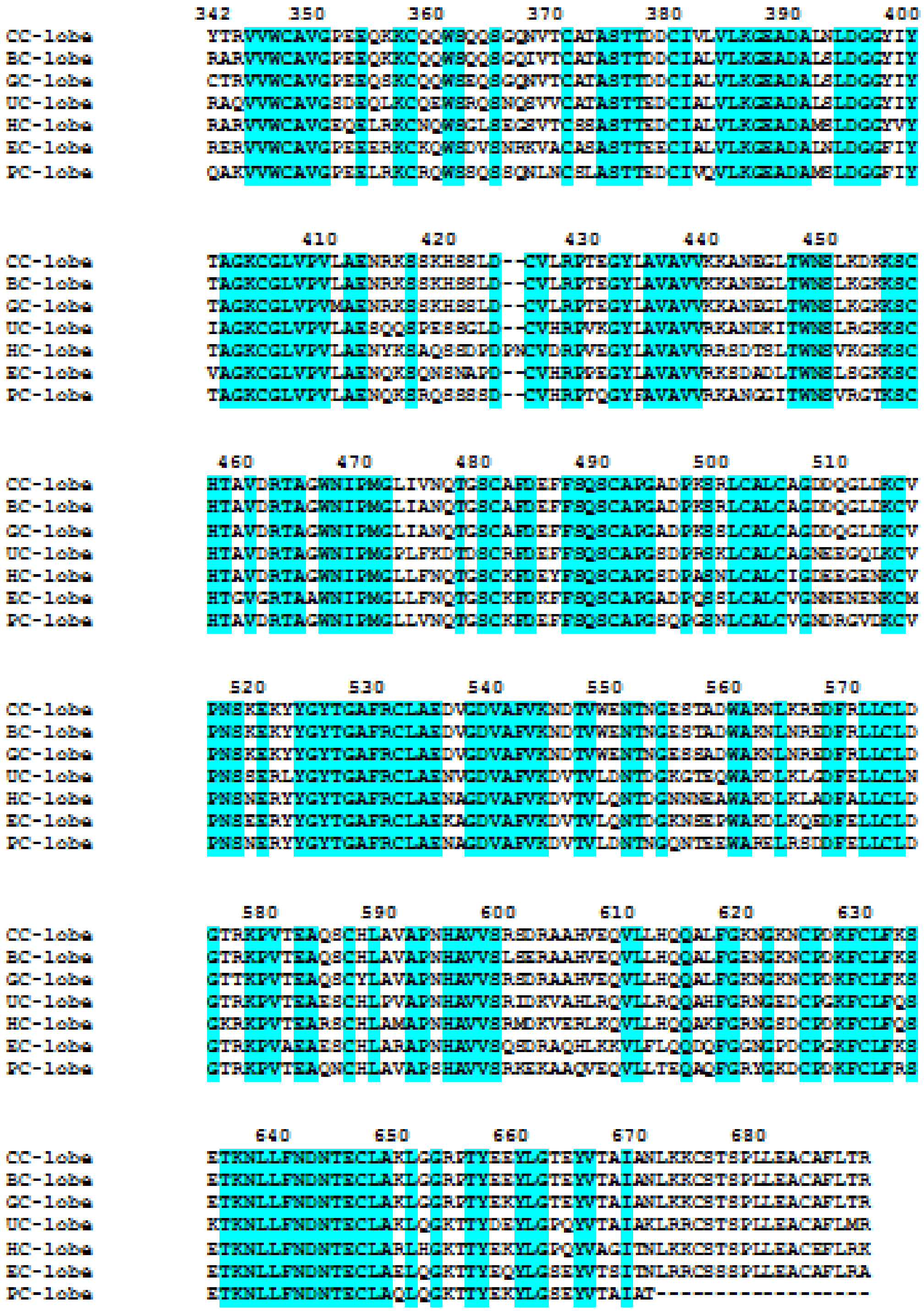

Fig. S3
